# Multi-tissue transcriptomics of the black widow spider reveals expansions, co-options, and functional processes of the silk gland gene toolkit

**DOI:** 10.1186/1471-2164-15-365

**Published:** 2014-06-11

**Authors:** Thomas H Clarke, Jessica E Garb, Cheryl Y Hayashi, Robert A Haney, Alexander K Lancaster, Susan Corbett, Nadia A Ayoub

**Affiliations:** Department of Biology, Washington and Lee University, Lexington, VA 24450 USA; Department of Biological Sciences, University of Massachusetts, Lowell, MA 01854 USA; Department of Biology, University of California, Riverside, CA 92521 USA; Department of Pathology, Beth Israel Deaconess Medical Center, Boston, MA 02115 USA; Center for Biomedical Informatics, Harvard Medical School, Boston, MA 02115 USA

**Keywords:** *de novo* assembly, Spidroin, Gene family, Molecular evolution, *Latrodectus hesperus*

## Abstract

**Background:**

Spiders (Order Araneae) are essential predators in every terrestrial ecosystem largely because they have evolved potent arsenals of silk and venom. Spider silks are high performance materials made almost entirely of proteins, and thus represent an ideal system for investigating genome level evolution of novel protein functions. However, genomic level resources remain limited for spiders.

**Results:**

We *de novo* assembled a transcriptome for the Western black widow (*Latrodectus hesperus*) from deeply sequenced cDNAs of three tissue types. Our multi-tissue assembly contained ~100,000 unique transcripts, of which > 27,000 were annotated by homology. Comparing transcript abundance among the different tissues, we identified 647 silk gland-specific transcripts, including the few known silk fiber components (e.g. six spider fibroins, spidroins). Silk gland specific transcripts are enriched compared to the entire transcriptome in several functions, including protein degradation, inhibition of protein degradation, and oxidation-reduction. Phylogenetic analyses of 37 gene families containing silk gland specific transcripts demonstrated novel gene expansions within silk glands, and multiple co-options of silk specific expression from paralogs expressed in other tissues.

**Conclusions:**

We propose a transcriptional program for the silk glands that involves regulating gland specific synthesis of silk fiber and glue components followed by protecting and processing these components into functional fibers and glues. Our black widow silk gland gene repertoire provides extensive expansion of resources for biomimetic applications of silk in industry and medicine. Furthermore, our multi-tissue transcriptome facilitates evolutionary analysis of arachnid genomes and adaptive protein systems.

**Electronic supplementary material:**

The online version of this article (doi:10.1186/1471-2164-15-365) contains supplementary material, which is available to authorized users.

## Background

High-throughput, next-generation sequencing allows for the efficient sequencing of millions of nucleotides from organisms lacking a reference genome [1, 2]. Next-generation sequencing therefore can identify numerous genes vital to key evolutionary innovations or unique adaptations in non-model organisms. One particularly robust tool for use in non-model organisms is deep sequencing of the mRNA, e.g., RNA-Seq. The depth of coverage afforded by the ability to sequence millions of RNA fragments, at a fraction of the time and cost of Sanger sequencing [3], facilitates *de novo* construction of transcriptomes (e.g. [4, 5]). The *de novo* transcriptomes can enable identification of functional genes without sequencing and assembling the often repetitive non-coding genomic regions (e.g., [6–10]). Additionally, sequencing mRNAs from specific tissues, developmental time points, or experimental conditions allows for rapid profiling of transcript abundance at a global scale [1] and analyzing phylogenetically restricted adaptations (e.g., social phenotypes in ants [10] and the capsaicinoid pathway in peppers [11]).

Spiders (Araneae) are a genome resource poor arthropod order, despite their taxonomic and ecological prominence. Araneae is one of the most species rich metazoan orders, consisting of over 44,000 described species that are found in every terrestrial ecosystem on the globe [12]. Spiders synthesize the most diverse repertoire of functionally differentiated silk fiber types among all the silk producing organisms. They are also by far the largest clade of venomous animals. The most closely related species with fully sequenced and annotated genomes diverged from spiders nearly 500 mya [13–16]. Unlike these fully sequenced arachnids, spiders are characterized by silk and venom production, the mechanics of which are poorly understood, due in part to the paucity of spider genomic resources. Recent studies have begun to use next generation sequencing in spiders to *de novo* assemble partial transcriptomes for a tarantula, an orb-web weaver [17], two cobweb weavers [18, 19], and three social species in the genus *Stegodyphus*[20]. These studies were restricted either to a single tissue (silk glands or venom glands, but not both) [17, 18] or whole animals [19, 20], limiting the biological interpretation of genes identified.

Silk synthesis is vital to spiders throughout all their life stages for numerous functions including prey capture, reproduction, protection, and dispersal [21]. Orb-web weaving spiders and their relatives (superfamily Orbiculariae) spin up to seven task-specific fibers and glues, each originating from different abdominal glands. Each of the functionally differentiated silk types has their own suite of remarkable material properties, including varying levels of stickiness, strength, stiffness and extensibility [22]. For instance, dragline silks synthesized in the major ampullate glands have tensile strength similar to steel, while capture spiral silk synthesized in flagelliform glands can stretch up to 300% [23]. The remarkable physical properties of silk have motivated attempts for *in vitro* synthesis of silk [24, 25], but these efforts have been hindered by a lack of understanding of the full molecular processes that create the silk fibers [26, 27].

Past molecular studies of silk have overwhelmingly focused on fiber-forming structural proteins (fibroins). The spider specific fibroins, also called spidroins, are encoded by members of a single gene family [28, 29]. Spidroin genes are known to be very large and internally repetitive (e.g., [30–33]) making them difficult to sequence and assemble *de novo*. Characterization of spidroins has largely been based on traditional Sanger-sequenced cDNA libraries. Each of at least six functionally differentiated silk types is formed from 1–2 distinct spidroins, which exhibit silk gland specific patterns of expression [28]. Within each silk gland type, the spidroins are highly expressed [34], which impedes the discovery of transcripts expressed in lower abundance, yet may nevertheless play important roles in silk production.

Recent studies have identified a few examples of non-spidroin genes involved in silk fiber and glue production. These include the egg case silk proteins, ECP-1 and ECP-2 [35], that appear to interact with the primary egg case silk spidroin TuSp1 [36]. However, ECP-1 and ECP-2 are expressed at levels that are several orders of magnitude below that of TuSp1 in tubuliform silk glands [37]. Additionally, a transcription factor, SGSF, has been implicated in the regulation of the egg case proteins in *Latrodectus hesperus*[38]. Finally, two non-spidroins expressed in the aggregrate silk gland have been shown to be involved in aqueous glue droplet production [39] and in web connection joints [40]. These various discoveries hint at a broad range of both regulatory and structural proteins involved with silk production.

Here we use *de novo* assembly of deeply sequenced cDNA fragments to characterize the transcriptome of the Western black widow, *Latrodectus hesperus* (Theridiidae). The Western black widow is an attractive spider with which to investigate the genomics of silk production given their strong dragline silk [41] and the existing molecular characterization of this species' spidroin encoding genes. *L. hesperus* has an estimated genome size of 1.3 billion bp [42], which while on the lower range of spider genomes, would be challenging to fully sequence. Thus far, spidroin paralogs have been described from five of the six functionally differentiated gland types in the black widow [33–35, 43, 44], including three of the six completely sequenced spidroin genes (*MaSp1*, *MaSp2*, and *AcSp1*[33, 34]). Of the spidroins synthesized by orb-weaving relatives of black widows, only Flag, the spidroin used in the orb-web capture spiral, remains unknown from *L. hesperus*, perhaps because *L. hesperus* builds a three dimensional cobweb that lacks the capture spiral. This collection of spidroin sequences can serve as a benchmark for the quality of the transcriptome and the capacity of the assembly to successfully integrate the large repetitive regions, where *de novo* transcriptome assembly has traditionally struggled [45, 46].

Our goal was to construct a high quality reference database that could be used to identify tissue specific expression patterns in black widows, and to contribute to ongoing evolutionary genomics of spiders. Here, we identify and analyze genes specifically expressed in silk glands, and thus represent candidates for silk components or involvement with silk synthesis, assembly or regulation. We evaluate the functions of these silk specific genes to generate a transcriptional program for silk glands. We also identify putative gene families to which these silk specific transcripts belong, allowing us to test if silk-restricted expression tends to evolve once, followed by gene expansions, or if, in contrast, silk-restricted expression has been co-opted from gene copies expressed in other tissues. More generally, our multi-tissue transcriptome is important for evolutionary analyses of any adaptive protein system present in spiders and provides extensive resources for the production of silk through recombinant or synthetic biology approaches.

## Results

### High quality black widow transcriptome

**Figure 1:**
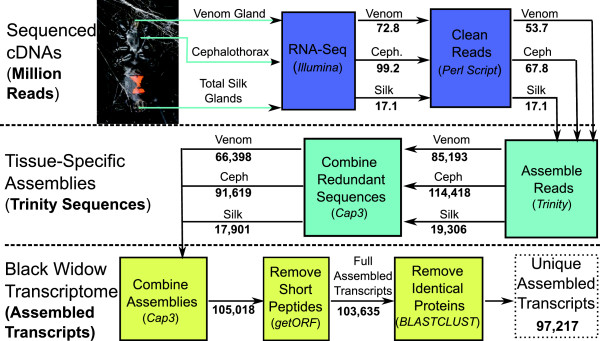
**Flowchart of the** ***de novo*** **transcript assembly process.** The Western black widow transcriptome was assembled in three major steps. First, high-quality 75 or 100 base paired-end cDNA sequence reads were generated for each of three tissues (dark blue boxes). Second, transcripts were *de novo* assembled for each tissue separately (light blue boxes). Finally, the high quality non-redundant transcriptome was generated (yellow boxes). Relevant programs are shown parenthetically in the boxes. The number of sequence reads, Trinity *de novo* assembled sequences, and final assembled transcripts generated in each step are shown in bold below the arrows.

We generated over 149 million, high quality, 75 or 100 bp paired-end sequence reads from genes expressed (cDNAs) in three tissues of adult female black widows: silk glands, venom glands and cephalothoraxes (Figure 1). *de novo* assembly of each tissue-specific library with Trinity resulted in ~19-115 thousand transcripts grouped into 16.8-72.1 thousand “components”, depending on the tissue type (Figure 1). A “component” is typically interpreted as representing a single genomic locus. Combining the three assemblies with CAP3 produced a multi-tissue transcriptome containing 103,365 sequences that are predicted to encode at least 30 amino acids (aa). All reads and the final transcriptome are available under BioProject accession PRJNA242358.

The multi-tissue transcriptome included complete homologs to 99% of the Core Eukaryotic Genes (CEGs), and the arthropod benchmarking genes (BUSCO). Importantly, the multi-tissue transcriptome recovered 99% of 999 previously described *L. hesperus* cDNA and genomic sequences. Based on BLASTX alignments to *Drosophila melanogaster* proteins, we found few potential cases of chimeric assembled transcripts (4.9%, E-score < 1e-50). In all of these metrics, the Trinity derived transcriptome outperformed an independently generated Velvet/Oases derived transcriptome (the comparison is described in detail in Additional file 1).

Trinity can have difficulty resolving allelic variants from isoforms or even paralogs, and thus errs on the side of splitting variants into separate transcripts [5, 45]. To account for this potential redundancy, we removed copies of assembled transcripts that were predicted to encode identical amino acid sequences, thereby reducing the total number of assembled transcripts from 103,635 to 97,217. Removing redundant assembled transcripts resulted in only a slight reduction of raw reads that aligned to the transcriptome, with 80-86% (depending on library) aligning to the initial set of assembled transcripts and 74-86% aligning to the Unique Assembled Transcripts (UATs, Additional file 2: Table S1). The non-redundant transcriptome still included 99% of the arthropod Benchmarking Universal Single Copy Orthologs (BUSCO [47], E-score < 1e-20). After removal of redundant amino acid sequences, 64% of the BUSCO genes matched multiple UATs in our transcriptome (mean UATs/ortholog = 8, median = 2, range = 0–174).

### Annotation of black widow transcriptome

**Figure 2:**
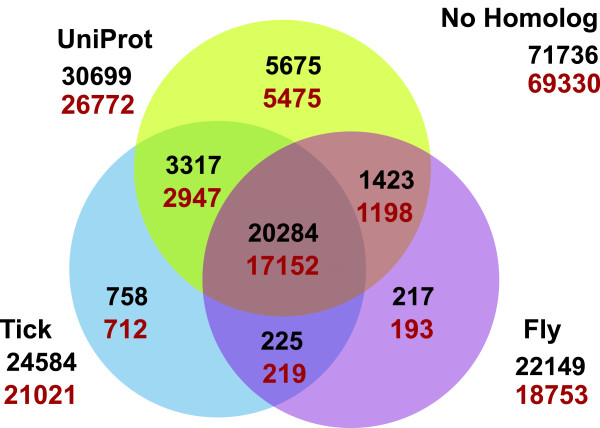
**BLASTX derived annotation of the Western black widow transcriptome.** The number of sequences with homology between the full set of assembled transcripts (shown in black) and the unique assembled transcripts (shown in orange) using E-score < 1e-5 to the UniProtKB database (yellow circle), fruit fly RefSeq proteins (pink circle), and the tick RefSeq proteins (blue circle) are shown.

Due to the lack of a closely related species with a well annotated genome, we approached the annotation of the assembled transcripts using a variety of methods. First, we used BLAST to ascertain homology to the reference proteins of one of the closest relatives with a completely sequenced and annotated genome, the deer tick *Ixodes scapularis*, and the reference proteins of the best annotated arthropod, the fruit fly *Drosophila melanogaster*, as well as a global protein database, UniProtKB. Approximately 30% of the transcriptome could be annotated by homology to tick, fruit fly, or UniProtKB (Figure 2). Second, since it is possible that our transcriptome contained sequences that are only partially complete, we looked for protein domains within the translated UATs using PFAM, though this only added annotations for an additional 0.5% of the UATs. Finally, we compared the translated UATs to the published *L. hesperus* proteins in GenBank (August 2013), but this annotated a scant additional 197 UATs (0.002%).

Deep sequencing can also reveal low-level contamination (e.g. lab bacterial strains, human) as well as infectious species (e.g. bacteria or fungi) or endosymbionts that are co-isolated with the mRNA of the species of interest. We identified potential non-black widow UATs using UniProt BLAST hits (Additional file 3: Figure S2). A sizable minority of the transcriptome was closest to bacterial genes (11.1% of annotated UATs). These potential contaminants contributed only a very minor proportion of the expression (0.2% of the total expression of the annotated transcripts as shown in Additional file 3: Figure S3). Examination of the genera of bacteria suggests that most of the bacterial sequences are not from endosymbionts, such as *Wolbachia*[48], but from lab strains, such as *E. coli* (Additional file 3: Figure S4). However, most of the expression is from neither endosymbionts nor *E. coli* (Additional file 3: Figure S3). These contaminants were removed from the published UATs in NCBI (see Additional file 4). There was negligible evidence of contamination from human genes (0.1%), similar to the number of “contaminants” from other model vertebrate species, including mouse, chimpanzee and frog, suggesting that these UATs are spider homologs of genes only described in vertebrates thus far.

### Functions of silk gland-specific transcripts

We were able to classify silk gland-specific transcripts (SSTs) by identifying UATs that had at least one expected count per million (eCPM) and were at least 631 times more abundant in silk compared to venom and 891 times more abundant in silk compared to cephalothoraxes. We used eCPM as opposed to correcting for sequence length (e.g., fragments per thousand base pairs per million mapped fragments, FPKM) so as not to discriminate against longer transcripts with low expression levels. These expression fold changes represented the top 0.5% most differentially expressed UATs between silk and the two other tissues (Additional file 3: Figure S1). Thus, out of 22,743 UATs with an eCPM > 1 in at least one tissue, 647 were silk gland specific, including 548 that were expressed exclusively in the silk glands. From the 647 SSTs, we found that 132 had a significant BLASTN hit (E-score < 1e-50) to our database of 999 non-redundant *L. hesperus* cDNA and genomic sequences. These 132 SSTs contributed 69.7% of the total silk gland expression, with nearly half of the total silk gland expression (48.8%) from 30 SSTs that matched spidroins. Approximately 8.8% of the expression in the silk glands is from the remaining SSTs.

To identify the functionality of the SSTs, we used homology to both the full proteins in UniProt and to domains in PFAM. Approximately 50% of the previously undescribed SSTs had a significant BLASTX alignment to a protein in UniProt or PFAM. Out of all the SSTs, we were able to assign GO terms to 25%, which is low compared to the non-SSTs with an eCPM > 1 (48%). The SSTs assigned GO terms based on UniProt were enriched for 14 GO terms and depleted for a single GO term compared to the entire set of UATs that had eCPM > 1 and were assigned GO terms (Additional file 5). The PFAM numbers were slightly lower as only 16% of the SSTs were assigned a GO term based on PFAM. There was a reduction in the PFAM GO terms in number enriched (5) and an increase in the number depleted (2). The enriched terms were a subset of the UniProt GO Terms, while the depleted terms did not overlap between PFAM and UniProt (Additional file 5).

#### SSTs are enriched in peptidases and peptidase-inhibitors

**Figure 3:**
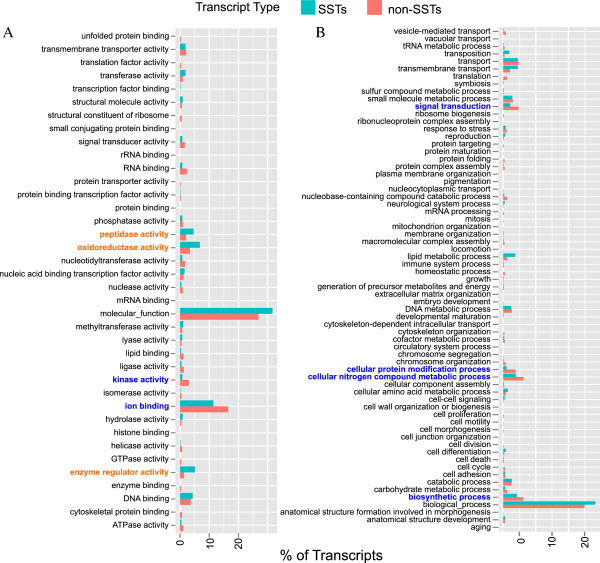
**GO SLIM term representation in both the silk specific transcripts (SSTs) and the non-SSTs.** Biological process **(A)** and molecular function **(B)** representation within SSTs (turquoise bar) and non-SSTs (orange bar) with eCPM > 1. GO terms that are significantly over-represented in the SSTs compared to all the UATs are bolded in dark orange while those that are significantly under-represented are shown in bold and blue. Significance was determined using a Wallenius test (FDR < 0.05).

The enriched and depleted GO terms within the SSTs implicated a wide range of functions. Paradoxically, the SSTs are enriched in both peptidase inhibitors (GO:0004867) and peptidases (GO:0008233), including several sub-sections of peptidases such as metallopeptidases (GO:0008237), and endometallopeptidases (GO:0004222). The SSTs are also enriched for functions related to oxygen, including oxidoreductase, oxidation-reduction, monooxygenase, dioxygenase, iron ion binding, heme binding, and choline dehydrogenase. The SSTs are depleted for ATP binding (UniProt), and nucleic acid and zinc ion binding (PFAM). Extending the analysis to GO SLIM terms, as opposed to the full GO set, reveals that the SSTs are depleted in the broad scale categories of signaling and binding proteins (Figure 3).

As spider silk proteins that are destined for incorporation into fibers must first be exported from the cell into the storage compartment, we searched for signal peptides at the N-terminal end of the translated UATs to identify potentially secreted proteins. From all the M-started predicted proteins in the non-redundant transcriptome with an eCPM > 1, we found that only 5.7% possessed a signal peptide according to SignalP [49]. In contrast, 26.2% of the M-started SSTs have signal peptides, representing a significant increase in expression of secreted proteins in silk glands compared to the entire transcriptome (Fisher’s exact test, *P* < 2.2e-16). We examined GO Terms of the UATs with signal peptides to see if the silk glands are secreting any functional class of proteins differently from the non-silk gland tissues. Most of the functions were not enriched for secretion in the SSTs compared to all of the transcripts, with the single exception of the peptidase inhibitors (GO:0010466). Of the SST peptidase inhibitors, 83% contain a signal peptide. In contrast, only 23% of the non-SST peptidase inhibitors contain a signal peptide (*P* = 0.08).

#### Silk-specific transcription factors

Silk glands are a spectacular example of gland-specific gene expression of the functionally differentiated spidroin gene family members. We thus expect to find silk gland specific transcription factors contributing to increased transcription of spidroins and other proteins that are incorporated into fibers in the silk glands. Using the UniProt-based GO Term annotation of site-specific transcription factor (GO:0003700), we identified ten SSTs, of which nine are a homolog of a fruit fly transcription factor (Table 1). As the transcriptional program of the fruit fly is well annotated, we were able to investigate the roles of fruit fly orthologs of our SST transcription factors. We observed that the orthologs of SST transcription factors are most highly expressed in a range of tissues and developmental stages, including the 8 hour embryo, the 16 hour embryo, L3 nerve cells and the embryonic S3 cell lines (Table 1). We also identified the known physical and genetic interaction partners of the SST transcription factor fruit fly orthologs collected in FlyBase. We then searched our black widow transcriptome for homologs of these interacting partners and, if found, determined if they were SSTs. We identified homologs of many of the fruit fly interacting partners (Table 1). However, none of the documented physical or genetic interaction partners of the SST transcription factor homologs in fruit fly had black widow homologs that were SSTs (Table 1).

**Table 1:** **Silk gland-specific unique assembled transcripts (UATs) predicted to encode transcription factors**

Western black widow SST ^a^	PFAM motif ID ^b^	Fruit fly homolog ^c^	Fruit fly tissue ^d^	Fruit fly interaction partners ^e^	UATs homologous to interaction partners ^f^
silk_Contig619	NA (homeobox)	FBgn0001235	L3 CNS	4	13 (0)
Contig373	Fork_head	FBgn0000659	6-8 hr Embryo	0	0
Contig303	T-Box	FBgn0261963	16 hr Embryo	0	0
silk_comp12525_c0_seq1	Homeobox	FBgn0000014	16 hr Embryo	2	6 (0)
Contig41	LIM	FBgn0026411	16 hr Embryo	0	0
silk_comp6942_c0_seq1	Homeobox	FBgn0003944	16 hr Embryo	20	64 (0)
Contig5112	TAFH	No match	NA	NA	NA
silk_comp16984_c0_seq1	Homeobox_KN	FBgn0001235	Larval stage 3 CNS cells	4	13 (0)
silk_comp16140_c0_seq1	Homeobox	FBgn0000015	S3 Cells	0	0
silk_comp15765_c0_seq1	Homeobox	FBgn0000015	S3 Cells	0	0

^a^SST = silk-specific transcript. See Methods for identification of SSTs.

^b^PFAM ID was assigned using highest match to a domain with HMMer 3.0.

^c^Fruit fly, *Drosophila melanogaster*, proteins were from the reference sequences (RefSeq) available in NCBI as of July 2012.

^d^Fruit fly tissues with the highest FPKM support were selected from FlyBase FPKM report obtained April 2013.

^e^Interaction partners are fruit fly genes noted in the FlyBase Physical Interaction Database obtained in April 2013 as interacting with the fruit fly homolog of black widow SSTs.

^f^Homologous black widow UATs to fruit fly interaction partners were chosen by best BLASTX match (SSTs in parentheses).

### Dynamics of silk-specific gene families

Thus far, only two gene families with silk gland-restricted expression have been identified, spidroins and egg case proteins (ECPs) [50]. The spidroins represent a relatively rapidly evolving gene family, in terms of sequence evolution, gland-specific expression, and functional diversification [28, 29, 44]. They also represent a gene family expansion that is both taxonomically (only known from spiders) and tissue (only known to be expressed in silk glands) restricted. To test if gene family expansion of genes expressed in silk glands is a general phenomenon, we constructed clusters of potential gene families with at least one member that was an SST. We found 12 putative gene families that had at least five SSTs, five of which were entirely composed of SSTs and seven of which included non-SST members (Table 2). The entirely SST families include one containing both the spidroins and ECPs (Family ID 5, 23 with best BLASTX to a spidroin, 5 with best BLASTX to an ECP). The largest SST-only cluster (27 members) contained 22 UATs with a significant BLASTX alignment to aggregate spider glue 2 from *Nephila clavipes* (Family ID 3, Table 2)*.* A third cluster composed of 12 SSTs includes an almost exact match (98.9% aa identity) to the recently described *L. hesperus* aggregate silk gland factor 2 (Family ID 19, Table 2). Two clusters contained 11 and 5 glycine-rich encoding transcripts, respectively, that had no significant identity to published proteins (Family IDs 24 & 49, Table 2). The final exclusively SST cluster contained 5 members with significant identity to a putative *L. hesperus* protein (Table 2). The clusters that included non-SSTs have a variety of functions including lipase, lipid transport, metalloproteolysis, and protease inhibition (Table 2).

**Table 2:** **Putative gene families that include at least five silk-specific transcripts (SSTs)**

Fam ID ^a^	# SSTs	# nSSTs ^b^	# eCPM < 1	Family description ^c^	UniProt ID ^c^
3	27	0	2	Aggregate spider web glue 2 (AgSG2)	B7SVM7
5	25	3	1	Egg case silk protein-1 (also spidroins)	Q52P73
7	8	9	7	Astacin-like metalloprotease	E7D164
9	9	13	0	Papilin	G0WRZ9
12	8	10	3	Serine protease inhibitor 28	G6DTM3
15	8	4	6	*L. hesperus* putative uncharacterized protein (Apolipoprotein from PFAM)	E7D1R3
16	13	2	2	*L. hesperus* putative uncharacterized protein (protease inhibitor from alternative UniProt)	E7D1V7
19	12	0	1	*L. hesperus* Putative uncharacterized protein (Aggregate gland Silk Factor 2^d^)	E7D1G4
24	11	0	1	No Annotation (Glycine Rich)	NA
45	5	2	0	*L. hesperus* putative uncharacterized protein	E7D1H3
49	5	0	1	No Annotation (Glycine Rich)	NA
65	5	0	1	*L hesperus* putative Uncharacterized protein	E7D1H1

^a^Family IDs were assigned to clusters generated by BLASTCLUST.

^b^Non silk specific transcripts (nSSTs).

^c^From BLASTX of UATs against UniProtKB, using the optimal hit in the family as decided by E-score.

^d^From BLASTP of translated UATs against published *L. hesperus* sequences.

#### Multiple derivations of silk-specific expression

Given that some of the putative gene families contain both SSTs and non-SSTs, we examined if the SSTs were each other’s closest relatives, and thus might represent a single evolutionary derivation of silk expression followed by gene family expansion within silk glands. Alternatively, silk-specific expression could have evolved at multiple times within the gene families. Using 35 gene families that had at least 2 SSTs and 2 non-SSTs (Additional file 2: Table S2), we calculated the ancestral states as either silk-specific or non-silk-specific using discrete Maximum Likelihood reconstruction. To increase our power of detection, we added transcripts as silk specific that had an eCPM > 1 and which were in the 2.5% tail for fold expression change, as opposed to the prior 0.5% (Additional file 3: Figure S1). This added 299 UATs to the set of potential SSTs, which were much more likely to group with a previously defined SST family than were other assembled sequences (26.1% versus only 1.5% of the remaining assembled sequences with an eCPM > 1). We then constructed 100 random trees for each of the 35 families and similarly calculated ancestral states. On average across the 35 families there are slightly fewer observed shifts from non-silk to silk-specific than expected from random trees (1.8 observed, 2.6 expected), but this is not significant in any of the trees (Additional file 2: Table S2). The two putative gene families in which there may be expansions of SSTs are Family ID 7, astacin-like metalloproteases (2 observed shifts from non-silk to silk-specific, 7.9 ± 2.7 expected) and Family ID 9, papilins (0 observed shifts from non-silk to silk-specific, 7.0 ± 2.8 expected).

#### Transcriptome reveals novel black widow spidroins

Gene families with expression restricted to silk glands can present obstacles for gene family reconstruction. For instance, because spidroins are highly repetitive and often contain simple (low complexity) amino acid sequence motifs, it is possible that non-homologous sequences can cluster according to similar amino acid compositions. Indeed, the ECPs are joined with the spidroins into a single cluster based on similar amino acid sequence motifs found in both families (e.g. runs of consecutive alanines or serines, doublets of glycine), even though ECPs lack the defining N and C-terminal domains of the spidroin gene family. Therefore, for each of the clusters that included previously described spider silk-specific proteins, we also used BLASTP derived alignments to break clusters into putative gene families.

**Figure 4:**
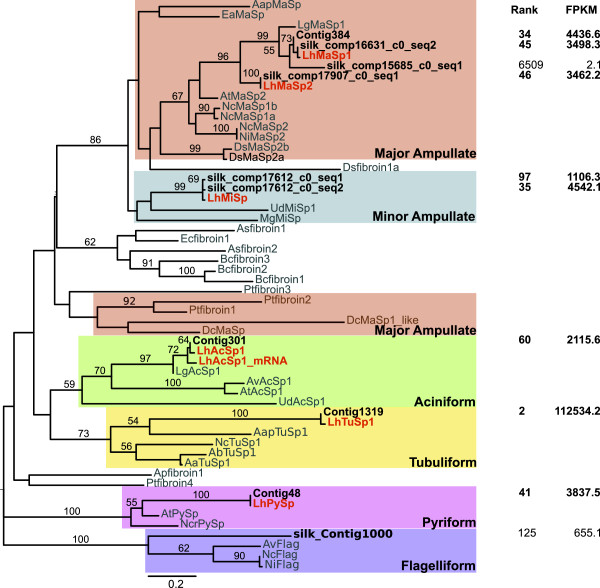
**Maximum likelihood tree of published spidroin C-termini and homologous black widow unique assembled transcripts (UATs).** The C-terminal regions of known *L. hesperus* (*Lh*) spidroins are shown in orange, other known spidroins are shown in grey, and translated UATs are black. Accession numbers for published spidroins are in [33, 44]. The tree is midpoint rooted and all bootstrap values greater than 50 are shown. Expression rank and FPKM (fragments per thousand base pairs per million mapped fragments) are shown on the right. Rank is based on FPKM in the silk glands out of all UATs expressed in silk glands. Clades corresponding to different gland specific expression are in colored rectangles.

**Figure 5:**
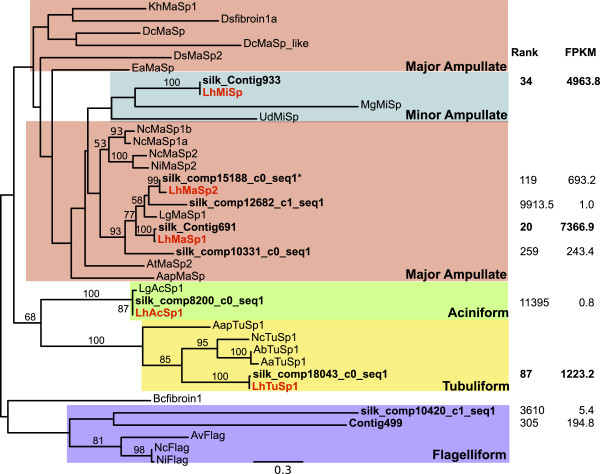
**Maximum likelihood tree of published spidroin N-termini and homologous black widow unique assembled transcripts (UATs).** The N-terminal regions of known *L. hesperus* (*Lh*) spidroins are shown in orange, other known spidroins are shown in grey, and translated UATs are black. Accession numbers for published spidroins are in [33, 44]. The tree is midpoint rooted and all bootstrap values greater than 50 are shown. Expression rank and FPKM (fragments per thousand base pairs per million mapped fragments) are shown on the right. Rank is based on FPKM in the silk glands out of all UATs expressed in silk glands. Clades corresponding to different gland specific expression are in colored rectangles.

Spidroins are expected to be difficult to *de novo* assemble due to their extreme length and repetitiveness. Nevertheless, our transcriptome included almost exact matches to the N and C-terminal encoding regions of all the described *L. hesperus* spidroins (Figures 4 and 5). These UATs included varying amounts of repetitive encoding sequence adjacent to the non-repetitive N or C terminal regions, although the C terminal containing fragments typically included more of the repetitive region than the N terminal containing fragments (amount of repetitive sequence adjacent to N- vs. C-terminal regions: 0–479 vs. 45–674; Additional file 2: Table S5). In addition to these almost exact matches to previously described *L. hesperus* sequences, we discovered new spidroin paralogs. Although multiple copies of *MaSp1* have been sequenced [34], we identified an additional MaSp1 C-terminal encoding UAT (silk_comp15685_c0_seq1; Figure 4) and N-terminal encoding UAT (silk_comp12682_c1_seq1; Figure 5). Each of these *MaSp1*-like UATs were only 74% identical to previously described *MaSp1* loci, compared to the other *MaSp1* UATs, which were 96-99% identical to previously described loci (Additional file 2: Table S5). However, these newly identified loci appear to be lowly expressed compared to the other *MaSp1* loci (Figures 4, 5). Likewise, we identified two UATs that grouped with the MiSp C-terminal region, one of which is 100% identical to the previously described *MiSp* mRNA, versus one that is only 85% identical over the entire length of the UATs (Additional file 2: Table S5).

Perhaps even more striking is the discovery of three UATs that grouped with Flag, the capture spiral spidroin, which was previously unknown in *L. hesperus.* One of the UATs, silk_Contig1000, strongly groups with Flag C-terminal domains (Figure 4). The other two UATs, Contig499 and silk_comp104020_c1_seq1, group with Flag N-terminal domains, albeit with low support (Figure 5). However, Contig499 is predicted to encode a complete protein that lacks characteristic spidroin amino acid motifs, although the most abundant amino acid is proline (11.2%), which is found in similar proportions in known Flag proteins. The other UAT that groups with the Flag N-termini, silk_comp104020_c1_seq1, only contains 11 aa predicted to be part of the repetitive region, which is insufficient to assess if this transcript encodes a protein with similar characteristics to previously described Flag repetitive regions. It is also possible that this UAT actually represents the PySp N-terminus, which has yet to be described in any spider.

#### Transcriptome reveals novel glue proteins

**Figure 6:**
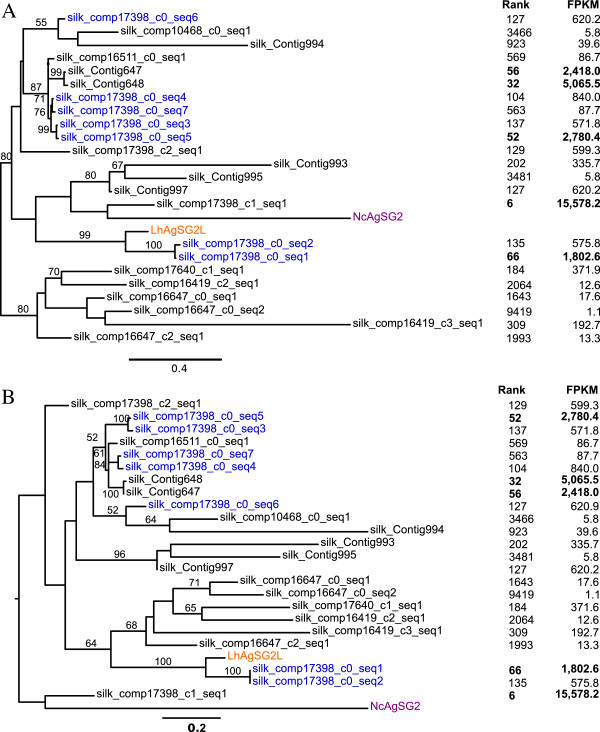
**Maximum likelihood tree of aggregrate spider glue 2 (AgSG2) and homologous unique assembled transcripts (UATs).** The trees were estimated from alignments of complete protein sequences **(A)** and domain-specific sequences **(B)**. Trees are midpoint rooted. Trinity-defined isoforms of a single component are shown in blue while the published *Nephila clavipes* (Nc) and published *Latrodectus hesperus* (Lh) sequences are shown in purple and orange, respectively. All bootstrap values greater than 50 are shown above branches. Expression rank and FPKM (fragments per thousand base pairs per million mapped fragments) are shown on the right. Rank is based on FPKM in the silk glands out of all UATs expressed in silk glands.

Thus far, no spidroins have been found expressed in the aggregate silk gland, which synthesizes aqueous glue droplets. Instead, a few distinct proteins have been described including Aggregate gland Spider Glue (AgSG) 1 and 2 from *Nephila clavipes*[39], and Aggregate gland Silk Factor (AgSF) 1 and 2 from *L. hesperus*[40]. We found 14 UATs that align significantly to *N. clavipes* AgSG1, but none of them are SSTs (Additional file 2: Table S3). In contrast, AgSG2 has a known homolog in *L. hesperus*[40], and we found 25 UATs with significant alignments to *N. clavipes* AgSG2, of which 23 are SSTs (Table 2, Figure 6). These AgSG2-like sequences range in length from ~140 to ~1200 aa. The shorter sequences all align to the same region of *N. clavipes* AgSG2 (aa positions 8–249), which is the most conserved region of the global alignment. Phylogenetic analyses of the global amino acid alignment as well as of the conserved region reveal multiple divergent clades, three of which include six of the top 60 most abundantly represented UATs in silk glands (Figure 6).

**Figure 7:**
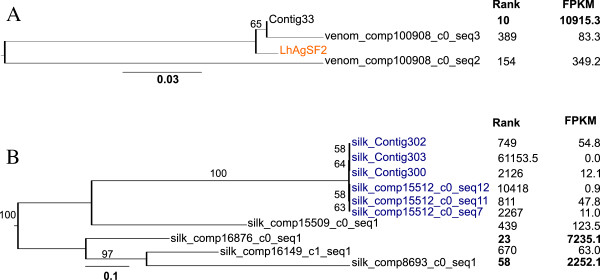
**Maximum likelihood trees of cluster containing black widow aggregrate gland silk factor 2 (AgSF2). (A)** Tree of unique assembled transcripts (UATs) that includes published *L. hesperus* (*Lh*) AgSF2 (orange). **(B)** Tree of translated UATs that clustered with UATs in (A) based on amino acid composition, rather than true homology. A third group of three UATs that was also in this cluster is not shown. Trinity derived isoforms of a single component are shown in dark blue. The trees are midpoint rooted. All bootstrap values greater than 50 are shown above branches. Expression rank and FPKM (fragments per thousand base pairs per million mapped fragments) are shown on the right. Rank is based on FPKM in the silk glands out of all UATs expressed in silk glands.

We also found seven UATs with significant alignments to AgSF1, six of which are in the top 100 most expressed sequences in silk glands (Additional file 2: Table S4). However, they did not group together using our clustering algorithm. Instead, these sequences likely represent fragments of the same gene. In contrast, we found a cluster of 13 sequences that included one that is almost an exact match to the previously published *L. hesperus* AgSF2 (Figure 7). Ten of the sequences in this cluster appear to be grouping with AgSF2 based on a high percentage of glycine, rather than true sequence identity. AgSF2 and the three UATs most similar to it (Figure 7A) are extremely glycine rich (27-41%) and the other ten UATs that clustered with AgSF2 are somewhat less glycine rich (11-21%). Both groups, however, include SSTs that are among the top 100 most abundantly represented sequences in silk glands (Figure 7A).

In both AgSG2 and AgSF2 families, there are multiple sequences associated with a single Trinity component, which is usually interpreted as evidence for isoforms generated from the same locus. For AgSG2-like, the isoforms are part of component 17398, and are either long sequences (silk_comp17398_c0_seq1 and seq2) of over 800 aa, or domain specific short sequences (silk_comp17398_c0_seq3-7). However, the conservation between the smaller sequences and the larger sequences makes it possible that Trinity is incorrectly joining these sequences into one component due to near identical sequences over at least 24 nucleotides. More likely, each of these UATs probably encodes paralogous small peptides, each containing portions of the AgSG2-like domain. An alternative pattern emerges in the AgSF2 cluster, where the six isoforms are identical, except for two indels of between 11 to 26 amino acids (Figure 7B).

## Discussion

Using deep sequencing of mRNAs expressed in three tissues, we generated a high quality transcriptome for the Western black widow. We captured 99% of the core eukaryotic genes (CEGs [51]), the benchmarking single copy orthologs in arthropods (BUSCO [47]), and previously described black widow genes (personal database). We were able to annotate 28,464 of the UATs by homology with published proteins (Figure 2). These sequences represent a minimum of 8149 unique protein-coding genes based on homology to unique tick proteins. Thus, compared to the currently published protein sequences in GenBank (414), we have increased the number of annotated Western black widow genes 20–69 fold, depending on the extent to which our ~28,000 annotated UATs represent paralogs versus alleles or isoforms. Our transcriptome represents a reference set for analyzing the evolution of spider genomes, identifying tissue-specific genes and their functions, and understanding the molecular processes underlying the evolution of novel spider protein-systems, such as silks (discussed here) and venom (discussed in [52]).

A large scale gene duplication event, such as a whole genome or chromosome duplication, in spiders was previously proposed based on the presence of two copies of each of the HOX genes in multiple spider species [53]. Consistent with this hypothesis, we found 64% of genes considered single copy in arthropods (BUSCO [47]) matched multiple UATs. Furthermore, we found an average of two homologs per core eukaryotic gene (CEGs). While some of our UATs may represent allelic variation, partially assembled genes, or isoforms, we expect a large proportion to represent genuine paralogous gene copies (e.g. Figure 6). Transcriptome or genome sequencing of additional spider species and phylogenetic comparisons with other arthropods will help elucidate whether spiders experienced a genome or chromosome-wide duplication event as opposed to smaller scale duplication events.

We focused on discovering candidate genes encoding silk components (fibers and glues) and genes involved in silk synthesis, fiber assembly, or regulating silk gland specific expression. The silk glands themselves present a number of obstacles to *de novo* assembly, including very few spider sequences available to use as a scaffold, and the potential for expression dominance of spidroins, which are very large and highly repetitive (e.g., [33, 34]). Additionally, we generated less than half the number of sequence reads for the silk glands and assembled fewer transcripts than the other tissues (Figure 1). Despite these limitations, we assembled near identical sequences to all the described genes known to have silk gland restricted expression, including the N-terminal, C-terminal, and portions of repetitive regions of spidroins (Figures 4 and 5), the complete Aggregate gland Spider Glues (Figure 6), and the complete Aggregate gland Silk Factors (Figure 7A). We additionally identified novel spidroin paralogs, most notably potential orthologs of the capture spiral protein, Flag, which had not previously been described for any cobweb weaving spider.

Overall, we identified 647 UATs with expression restricted to silk glands or considerably higher in silk glands than other tissues, which we have designated SSTs (silk-specific transcripts). Many (75%) of the SSTs were not assigned a functional annotation by association with a Gene Ontology (GO) term, possibly because they represent silk fiber or glue components that have not been assigned GO terms (e.g. spidroins and aggregate gland glues do not have GO terms). However, based on the GO terms that were assigned to the SSTs, we found enrichment for both proteinases and proteinase inhibitors, and a number of functions involved in oxidation or oxidation-reduction (Figure 3, Additional file 5). We thus propose that the primary roles of the silk glands are to synthesize fiber (e.g., spidroins) and glue components, transport these components out of the cell, protect these proteins from degradation while in the storage compartment, and then assemble and extrude these fibers and glues. Under this model, the seemingly paradoxical dual enriched terms of serine peptidase inhibitor and protease, can be explained by preferential exportation of peptidase inhibitors out of the cell. The high proportion of SST peptidase inhibitors with a signal peptide supports this hypothesis. The peptidase inhibitors can protect the spidroins against proteolysis in the external cellular environment, while, within the cell, the proteases can be used to degrade all non-exported, and therefore failed, spidroins or other proteins. The enrichment of GO terms associated with oxidation-reduction is consistent with the fact that silk proteins, at least in major ampullate silk glands, undergo a number of pH changes as they travel through the duct that aid in fiber assembly [54].

Spider silk glands are especially notable due to the evolution of morphologically distinct glands that synthesize functionally differentiated spidroin paralogs [28, 29]. Because we profiled the combined set of silk gland types, we are unable to propose novel silk gland type-specific genes or evaluate the extent to which a single spidroin paralog is exclusively expressed in a single gland type. We were also limited in discovering transcription factors that could regulate the gland-specific expression of spidroins and other fiber or glue components. All the same, we discovered ten SSTs that were putative transcription factors (Table 1). Gland-specific expression profiling, using our transcriptome as a reference, will help elucidate if these transcription factors are integral to the regulation of spidroins and/or glues. Intriguingly, the SST transcription factor homologs in *Drosophila melanogaster* are involved in regulating genes in a variety of tissues and developmental stages (Table 1), suggesting that potential silk gland regulatory systems were derived from multiple tissue types rather than co-opting an entire tissue system en masse.

Our transcriptome enabled us to address questions about the extent to which silk gland functions evolved through gene family evolution. The spidroins form the paradigm for the evolution of silk gland restricted paralog expression. The spidroins are a spider-specific and highly dynamic gene family that evolved through gene duplication and sequence and expression divergence. The expansion of this gene family concomitant with the morphological differentiation of the silk glands has been proposed as the explanation for the evolution of novel silk functions [28, 29, 33, 44]. However, the putative families of SSTs we describe offer additional models for silk specific functional expansions. One alternative model to gene family expansion is to generate multiple isoforms from a single gene. This possibility was observed in the clusters of assembled sequences with homologs to previously described aggregate gland-specific genes where the genes shared the same Trinity-derived identifier (Figures 6 and 7). However, within these families, it is also possible that the Aggregate gland Spider Glue 2 homologs are simply difficult for Trinity to assemble given their high level of sequence identity within a conserved domain. Even accounting for the potential to generate multiple isoforms from a single gene, our phylogenetic analyses demonstrate multiple divergent sequences are homologous to Aggregate gland Spider Glue 2 (Figure 6), suggesting gene family expansion. If this gene family is restricted in expression to the aggregate gland it will represent a markedly different pattern from the spidroin gene family, in which gene duplication often co-evolves with glandular differentiation. Aggregate glands are often enormous compared to the other silk glands, and gene family expansion may expedite the synthesis of copious amounts of aggregate glues.

An additional alternative model to the spider silk gland-specific gene family expansion is the independent derivation of silk gland-restricted expression from paralogs expressed in other tissues. The clustering of many SSTs with non-SSTs supports this model. In contrast to the spidroins and glue proteins, these putative gene families are not restricted to spiders (Table 2). In most cases of SSTs clustering with non-SSTs, we did not find evidence for single derivations of SSTs (e.g., SSTs did not group together within these clusters). Instead, SSTs were interspersed with non-SSTs, suggesting recurrent co-option of paralogs of non-silk specific genes within the silk glands. Two potential exceptions were clusters containing members homologous to (1) astacin-like metalloproteases and (2) papilins, which are metalloprotease inhibitors. Within these putative gene families, there were many fewer shifts from non-SST to SST, compared to the random expectation (Additional file 2: Table S2), suggesting potential gene expansions within silk glands. Considering the importance of protecting spidroins from degradation in the storage compartment, it is possible that the proteases and protease inhibitors co-evolved with spidroin paralogs.

## Conclusions

Silk fibers are vital to the lifetime fitness of spiders. The spectacular mechanical properties of spider silks motivate the development of numerous biomimetic applications. Our *de novo* transcriptome provides an illuminating glimpse into the functional and evolutionary processes involved in silk production, as well as resources for further investigations into silk glands and other spider tissues. While the spidroins have historically occupied the central space within the study of silk glands, our analyses indicate a rich transcriptional program beyond the spidroins, including multiple functional roles of non-spider specific genes, new models of gene family expansion in spider specific genes, and multiple derivations of silk-specific expression from closely related paralogs expressed in other tissues. The efficiency of generating our transcriptome demonstrates the lowered barriers to performing genomic analysis even within species and tissues currently lacking any reference sequences. Our findings strongly support expansion of genomic resources and analyses of the functionally differentiated silk gland types, other tissues of interest, as well as into other spider species.

## Methods

### *L. hesperus*transcriptome assembly

#### Sampling, dissections, RNA isolations, library construction

Adult female black widows were collected in Riverside (Riverside County, California, USA) in March 2009 and July 2010. The subject of our study, the Western black widow spider *Latrodectus hesperus*, is an unregulated invertebrate, as *L. hesperus* is neither threatened nor endangered. Total RNA was isolated from the combined silk glands of a single individual, the cephalothorax of another individual (with the venom glands removed), and seven pairs of venom glands. RNA was extracted from homogenized tissue in TRIzol^**®**^ (Invitrogen) and further purified with the RNeasy kit (Qiagen). Potentially contaminating DNA was removed with Turbo DNase (Ambion).

cDNA libraries were prepared for sequencing with the mRNA sequencing sample preparation kit (Illumina, San Diego, CA). In brief, poly-A mRNA was isolated with two rounds of treatment with oligo(dT) magnetic DynaBeads. The mRNA was randomly fragmented by heating to 94°C in fragmentation buffer. First strand cDNA was synthesized with SuperScript^**®**^ III reverse transcriptase (Invitrogen) primed with random hexamers. Second strand cDNA was synthesized by incubation with RNase H and DNA Pol I. Double stranded cDNAs were end repaired, A-tailed, and ligated to Illumina “PE adapters”. Discrete sized cDNA-adapter ligation products of 350–500 base pairs (depending on library) were selected by electrophoresis and purified from agarose gel slices using the QiaQuick Gel Extraction Kit (Qiagen). cDNA templates were enriched by 15 cycles of PCR with Phusion polymerase (New England Biolabs). The silk library was sequenced with 75 paired-end cycles on a single lane of the Genome Analyzer I and the cephalothorax and venom libraries were sequenced with 100 paired-end cycles in separate lanes of the Genome Analyzer II (Illumina).

#### *de novo*transcript assembly

Prior to assembly, FASTQ files generated by Illumina sequencing were processed to remove any adapter or low quality sequences (we trimmed reads with a quality score of less than 28 from the end of each read, and removed entirely both read mate-pairs for which this procedure resulted in a sequence less than 60 nucleotides from either of the read mate-pairs). The FastQC package [55] was used to verify the quality of the resulting trimmed and filtered reads. Transcripts from each tissue-specific library were *de novo* assembled separately using Trinity [5] with default parameters. We compared the output of Trinity to another *de novo* assembly program, Velvet-Oases [56] (Additional file 1).

To generate the most complete possible set of *L. hesperus* transcripts we combined tissue-specific assemblies using CAP3 (Figure 1). We first ran CAP3 using default parameters on each tissue specific assembly and labeled the resulting contiguous sequences (contigs) and singletons according to tissue type. We then concatenated all six files (tissue-specific contigs and tissue specific singletons) and again ran CAP3 with default parameters. Assembled transcripts generated from combining tissue-specific assemblies thus do not retain any tissue-specific labeling. We predicted open read frames (ORFs) for each of the resulting assembled transcripts using GetOrf [57] and retained only those that were predicted to encode at least 30 amino acids. These sequences represent the full set of assembled transcripts.

To generate our predicted proteins, we translated assembled transcripts using the frame of the best hit to NCBI’s nr database, if a BLASTX hit was available. If not, the longest open reading frame (ORF) was identified and used to predict the amino acid sequence. In cases where the longest ORF had a stop codon both in the 5′ region and in the 3′ region and the length of the ORF from the first M onwards was at least 75% of the length of the total ORF, the first M was used as the starting position for the predicted protein.

The predicted proteins were used to remove redundant protein-encoding transcripts from the transcriptome. These were identified using BLASTCLUST [58] to group amino acid sequences that were identical over the full length of the shorter member. For each resulting cluster, the longest amino acid sequence was chosen as the representative. In cases where multiple transcripts had identical optimal lengths, the first optimal transcript in the cluster was picked. The representative cluster members were combined with remaining unique protein-coding sequences to form the non-redundant transcriptome, or set of unique assembled transcripts, UATs (Figure 1).

#### Transcriptome assessment

To determine the quality/accuracy of our Western black widow transcriptome, we first compared our assembled transcripts with previously characterized *L. hesperus* cDNA and genomic sequences compiled from (1) all nucleotide sequences downloaded from NCBI’s nt and dbEST databases (January 2012), and (2) a personal database of unpublished cDNA and genomic sequences. We made a non-redundant set of (1) and (2) using CAP3 with default parameters. We then created a BLAST database of our transcriptome and aligned the known *L. hesperus* nucleotide sequences using BLASTN [59] with an E-score cutoff of 1e-50 to determine how well the transcriptome recovered known sequences. We also compared our assembled transcripts to two benchmarking datasets: (1) a database of orthologous genes found in all eukaryotes with sequenced genomes using CEGMA [51]; (2) the arthropod Benchmarking set of Universal Single-Copy Orthologs (BUSCO) [47] using TBLASTN with an E-score cut off of 1e-20. To further identify arthropod-specific genes we compared our assembled transcripts to the reference set of proteins from the deer tick, *Ixodes scapularis* (v1.2), and the fruit fly, *Drosophila melanogaster* (v5.3), using BLASTX with various E-score cutoffs. We used the BLASTX results with the fruit fly proteins to determine the proportion of assembled transcripts that were potentially chimeric combinations of sequences that were generated from different genes. If the top 20 BLASTX hits included different protein IDs and those multiple proteins aligned to different regions of the same assembled transcript (alignments could only overlap for at most 10 bases) then the assembled transcript was considered a potential chimeric.

In addition to alignments to Core Eukaryotic Genes, BUSCO, tick, and fruit fly, we also aligned our assembled transcripts to proteins in UniProtKB (August 2012 release) and NCBI’s nr database (August 2012) using BLASTX with E-score cutoffs of 1e-5. We additionally aligned predicted proteins to the PFAM database using HMMer version 3.0.

To further characterize functions of transcripts with homology to known proteins, we obtained the Gene Ontology (GO) terms [60] associated with both the best UniProt and PFAM hits as determined by E-score for each non-redundant transcript. GO SLIM terms were obtained using the program GO SLIM Viewer [61]. Additionally, taxonomic information for each transcript was derived using the taxonomic identification of the optimal UniProt BLAST hit as decided by lowest E-score. GO and GO SLIM terms significantly enriched in subsets of transcripts compared to the entire set were identified using the GoSeq R package [62] with the Wallenius and the HyperGeometric tests.

Proteins that are secreted out of the cell tend to have characteristic signal peptides. These were ascertained using SignalP v 4.0 [49] using the longest M-started ORFs for each of the transcripts where the ORF contained at least one M. All ORFs entirely lacking M were not searched for signal peptides.

### Identifying silk gland-specific transcripts

Transcript abundance in silk glands, cephalothorax, and venom glands was estimated by aligning the processed raw paired-end sequence reads from each tissue-specific library to the final non-redundant transcriptome using RSEM [63]. RSEM provides an estimate of number of sequence reads that originated from a given transcript, accounting for the possibility that a single read could align to multiple transcripts. Once the expected counts of each transcript was estimated by RSEM, we accounted for differences in tissue-specific library size by calculating the expected counts per million aligned reads (eCPM) for each UAT in our non-redundant transcriptome. We then removed all UATs with an eCPM less than one in all libraries.

To identify UATs potentially involved in silk production we identified those that were uniquely expressed in silk glands (>1 eCPM in silk, 0 eCPM in other tissues) as well as those that were much more abundant in silk glands than in cephalothoraxes or venom glands. For the latter, we calculated the ratio of eCPM for silk: venom and silk:cephalothorax. We chose as silk-specific transcripts (SSTs) those UATs that showed a fold change greater than 99.5% of other UATs (Additional file 3: Figure S1).

### Gene family evolution of silk gland-specific transcripts

Initial clusters of SSTs that could represent gene families were constructed by running BLASTCLUST on amino acid sequences predicted from the SSTs using default values except allowing for 50% identity over 50% of the shorter sequence. Non-silk-specific UATs were added as cluster members if they had BLASTP alignments to at least one member of a silk-specific cluster with at least 50% identity over 50% of the shorter amino acid sequence.

Gene trees were constructed for clusters containing at least two UATs with a silk:cephalothorax and silk: venom fold change in the top 2.5% tail and two other UATs. Amino acid alignments were generated using MUSCLE [64] and phylogenetic trees were constructed with PhyML [65] using default values for both, except for using the JTT substitution model in PhyML. The trees were then midpoint rooted. Internal nodes were labeled as either silk gland-specific or non-silk-specific using maximum likelihood ancestral state reconstruction implemented with the APE r-package v 3.1 [66] using the default parameters for discrete states. For nodes where there was an equal likelihood of being in either state, these were classified as non-silk. Rooted random trees were constructed using the rooted tree creator within APE with the number of UATs in the gene family as the input parameter. UATs were assigned to the leaves of the random trees and the interior nodes were labeled as before.

Additional gene trees were estimated for families of UATs that aligned to genes previously known to have silk gland-specific functions including spidroins, Aggregate gland Spider Glue 2 (AgSG2), and Aggregate gland Silk Factor 2 (AgSF2) as determined through BLASTX. In the case of spidroins, only the non-repetitive N and C-terminal regions can be used for phylogenetic reconstruction due to the inability to determine positional homology in the rapidly evolving repetitive regions [44]. UATs encoding spidroin N or C-terminal regions were identified by BLASTX or TBLASTN alignments to previously characterized spidroin N and C-termini (excluding repetitive regions). The UATs were added to separate alignments of nucleotides encoding N and C-termini generated by Garb et al. [44] and Ayoub et al. [33]. UATs were translated, repetitive regions removed, and then aligned using MUSCLE followed by manual adjustment. Published AgSG2 sequences (*Nephila clavipes*, GenBank:AFP57559, and *L. hesperus* GenBank:AFP57559) were added to the alignment of the cluster identified by BLASTCLUST that contained 22 members with significant BLASTX alignments to these sequences. The published AgSF2 (*L. hesperus*, GenBank:AFP57562) was added to the cluster that contained members with a significant BLASTX hit to this sequence. Both aggregate gland glue and silk factors were aligned using the BLASTP results as a seed followed by MUSCLE global alignment.

### Availability of supporting data

All reads and the final transcriptome described in the manuscript are available under BioProject accession PRJNA242358.

## Electronic supplementary material

Additional file 1: **Creation and Comparison of the Trinity and the Velvet/Oases Transcript Assemblies.** A prose description of the creation and comparison of the Trinity and Velvet/Oasis assemblies with the number of sequences assembled (Additional file 1: Table S1) and a side-by-side metric comparison between the two (Additional file 1: Table S2). (PDF 122 KB)

Additional file 2: **Additional resources for the consequences of removing non-unique transcripts (Table S1), silk gland-specific gene family evolution (Table S2), and homologs of spider-specific genes (Tables S3-S5)**. (PDF 131 KB)

Additional file 3: **Additional resources for identifying possible bacterial contamination (Figure S2-4) and silk gland-specific transcript identification (Figure S1,S5).** (PDF 214 KB)

Additional file 4: **List of all Unique Assembled Transcripts and their Attributes.** (ZIP 2 MB)

Additional file 5: **GO and GO SLIM Annotation.** A table showing the GO term annotation of both silk gland-specific transcripts (SSTs) and non-silk gland specific transcripts (nSSTs) using both the full GO and the GO SLIM annotations based on UniProtKB and PFAM homology. (XLSX 399 KB)

## Abbreviations

aa: Amino acids
AgSF: Aggregate gland Silk Factor
AgSG: Aggregate gland Silk Glue
bp: Base pairs
BUSCO: Benchmarking Universal Single Copy Orthologs
CEG: Core eukaryote genes
ECP: Egg case protein
eCPM: Expected counts per million reads
FPKM: Fragments per kilobase per million reads
GO: Gene Ontology
MaSp: Major Ampullate Spidroin
ORF: Open Reading Frame
SST: Silk gland-specific transcript
TuSp: Tubuliform Spidroin
UATs: Unique assembled transcripts.
