# Supplementary material for: Multi-tissue transcriptomics of the black widow spider reveals expansions, co-options, and functional processes of the silk gland gene toolkit
Source: BMC Genomics. 2014 Jun 11;15(1):365. doi: 10.1186/1471-2164-15-365 (PMC4200122; doi:10.1186/1471-2164-15-365)
Supplement: Supplementary file 1 — Additional file 1: Creation and Comparison of the Trinity and the Velvet/Oases Transcript Assemblies. A prose description of the creation and comparison of the Trinity and Velvet/Oasis assemblies with the number of sequences assembled (Additional file 1: Table S1) and a side-by-side metric comparison between the two (Additional file 1: Table S2). (PDF 122 KB) [file 12864_2013_6107_MOESM1_ESM.pdf]

Supplementary Methods

de novo transcript assembly

Transcripts were *de novo* assembled using Velvet-Oases [1] and Trinity [2]. Velvet [3] is intended to assemble whole genomes and the algorithm expects equal sequencing read depth across the entire assembly, though mRNA abundance can vary widely. In addition multiple transcripts (isoforms) can be generated from a single locus. Oases [1] attempts to assemble all isoforms and deals with unequal read depth. Trinity is a stand along package built for *de novo* assembly of mRNA-sequencing data.

Each tissue-specific library was assembled separately. Velvet-Oases was run with multiple k-mers ranging from 29-61 in increments of 4 using the Oases supplied Python script. The multiple runs were merged with the Oases –merge option. Because Velvet-Oases generated 30 – 400 thousand transcripts depending on library, a single "transcript" per "locus" was chosen using the oases-to-csv python script [4]. Trinity was run with default parameters using a single k-mer of 25.

To generate the most complete possible set of *L. hesperus* transcripts we combined tissue-specific assemblies using CAP3 [5]. We first ran CAP3 [5] using default parameters on each Trinity-derived tissue specific assembly and labeled the resulting combined sequences (or contigs) and singletons according to tissue type. We then concatenated all six files (tissue-specific contigs and tissue specific singletons) and again ran CAP3 with default parameters. For Velvet-Oases derived assemblies, we chose the "best" transcript for each "locus" using the oases-to-csv python script [4] for each tissue-specific assembly. We then ran CAP3 with default parameters on three concatenated "best transcript" files. Contigs generated among libraries do not retain any tissue-specific labeling. We predicted open read frames (ORFs) for each of the resulting assembled transcripts from both programs using GetOrf [6] and retained only those that were predicted to encode at least 30 amino acids. We compared the quality and completeness of the Trinity-derived transcriptome to the Velvet-Oases derived transcriptome by comparison to previously described proteins, according to methods described in the main document.

Results

Trinity derived transcriptome out performs Velvet-Oases

We generated over 149 million high quality 75 or100 bp paired-end sequence reads from genes expressed (cDNAs) in three tissues of adult female black widows, silk glands, venom glands and cephalothoraxes (Additional File 1, Table S1). *de novo* assembly of each tissue-specific library resulted in 19-450 thousand transcripts depending on assembly method and tissue type (Additional File 1, Table S2). These transcripts were grouped into “loci” or “components” by Velvet-Oases [1] and Trinity [2], respectively. “Loci” and “components” have similar underlying mathematical definitions and are typically interpreted as representing the same genomic locus. Multiple transcripts (e.g. isoforms) can be generated from a single locus. Trinity assemblies resulted in more loci (16.8-72.1 thousand) than Velvet-Oases (10.6-36.5 thousand), but fewer total transcripts (Additional File 1, Table S1; Trinity: 19.3-114.4 thousand, Velvet-Oases: 36.7-426.7 thousand). Due to the large numbers of transcripts generated by Velvet-Oases, we used a single transcript per locus for combining the tissue-specific assemblies into a putative transcriptome using CAP3. We retained all transcripts for combining tissue-specific assemblies into a Trinity derived transcriptome.

The Trinity derived assembly was more complete than the Velvet-Oases derived assembly in terms of possessing more homologs to a number of sets of previously described sequences. For instance, the Trinity derived transcriptome included complete homologs off 99% of the Core Eukaryotic Genes (CEGs), while Velvet-Oases recovered 90% of CEGs, as determined by CEGMA [7]. The Trinity derived transcriptome also possessed homologs of more unique tick and fruitfly RefSeq proteins than did Velvet-Oases assessed by significant BLASTX alignments (E-score < 1e-5; Table S2). Importantly, the Trinity derived transcriptome recovered 99% of 999 previously described non-redundant *L. hesperus* cDNA and genomic sequences while the Velvet-Oases transcriptome only recovered 88% (Additional File 1, Table S2). Finally, using BLASTX alignments to tick proteins, we found fewer potential cases of chimeric “assembled sequences” in the Trinity derived transcriptome than the Velvet-Oases derived one. Specifically, 11.2% of Trinity derived assembled transcripts had non-overlapping alignments to two different fruit fly proteins versus 13% of Velvet-Oases derived ones (E-score < 1e-10). Using more stringent alignments (E-score < 1e-50), only 4.9% and 6.7% of assembled transcripts were potentially chimeric in the Trinity and Velvet-Oases derived transcriptomes, respectively.

References

1. Schulz MH, Zerbino DR, Vingron M, Birney E: **Oases: robust *de novo* RNA-Seq assembly across the dynamic range of expression levels.** *Bioinformatics* 2012, **28**(8)**:**1086-92.

2. Grabherr MG, Haas BJ, Yassour M, Levin JZ, Thompson DA, Amit I, Adiconis X, Fan L, Raychowdhury R, Zeng Q, Chen Z, Mauceli E, Hacohen N, Gnirke A, Rhind N, di Palma F, Birren BW, Nusbaum C, Lindblad-Toh K, Friedman N, Regev A: **Full-length transcriptome assembly from RNA-Seq data without a reference genome.** *Nat Biotechnol* 2011, **29**(7)**:**644-52.

3. Zerbino DR, Birney E: **Velvet: algorithms for *de novo* short read assembly using de Bruijn graphs.** *Genome Res* 2008, **18**(5)**:**821-9.

4. **Oasis to CSV** [https://code.google.com/p/oases-to-csv/]

5. Huang X, Madan A: **CAP3: A DNA sequence assembly program.** *Genome Res* 1999, **9**(9)**:**868-877.

6. **EMBOSS GetOrf** [http://emboss.sourceforge.net/apps/cvs/emboss/apps/getorf.html]

7. Parra G, Bradnam K, Korf I: **CEGMA: a pipeline to accurately annotate core genes in eukaryotic genomes.** *Bioinformatics* 2007, **23**(9)**:**1061-7.

Supplementary Tables for Additional File 1

Table S1. Summary stats of Trinity (T) and Velvet-Oases (O) assemblies.

			Loci with Transcript Number											# components / Total transcripts	Contigs/ Singletons after CAP3
	PE[a]		1	2	3	4	5	6	7	8	9	10	>10		
Silk	17.1	T	15588	900	143	108	43	22	10	7	5	9	26	16835/ 19306	1068/ 16833
		O	6491	916	692	449	363	325	230	206	169	129	624	10594/ 36658	NA
Ceph	67.8	T	57650	7308	2925	1658	883	660	411	308	208	167	584	72178/ 114418	13942/ 77677
		O	16730	3450	2172	1611	1421	1236	1040	982	994	718	6168	36522/ 426672	NA
Venom	53.7	T	39305	5780	2506	1476	831	567	345	221	179	126	416	51336/ 85173	11616/ 54782
		O	16277	2495	1774	1305	1112	979	778	786	701	655	5516	32378/ 292812	NA

[a]Millions of processed paired-end reads used for *de novo* assemblies.

Table S2. Comparison of *de novo* assembly methods for black widow RNA-seq reads from silk glands, cephalothoraxes, and venom glands.

	[a]Trinity	[a]Velvet-Oases
# assembled transcripts (ATs)	103,635	53,644
N50	1554	1437
N90	273	316
[b]# ATs that aligned to fruit fly, BLASTX 10^{-05}	19374	7241
[b]# ATs that aligned to fruit fly, BLASTX 10^{-50}	8793	5880
[c]# ATs that aligned to tick, BLASTX 10^{-03}	24,584	15,493
[c]# ATs that aligned to tick, BLASTX 10^{-50}	9513	5779
[d]# unique tick proteins aligned to AT, BLASTX 10^{-05}	7900	7241
[d]# unique tick proteins aligned to AT, BLASTX 10^{-50}	4257	3876
[e]Potential Chimerics (BLASTX 10^{-10})	11.2%	13.0%
[e]Potential Chimerics (BLASTX 10^{-50})	4.9%	6.7%
[f]CEGMA % complete	98%	90%
[f]CEGMA % partial	99%	96%
[f]average number of complete homologs per CEG	2.06	2.01
[f]percentage of detected CEGS that have more than 1 homolog	52	58
[f]total number of CEGs present including putative paralogs	501	446
[g]Proportion of previously described black widow cDNAs and genes that aligned to an AT, BLASTN e^{-50}	99%	88%

[a]Assemblies were performed on three tissue-specific libraries separately and then combined using CAP3.
[b]Fruit fly, *Drosophila melanogaster*, proteins were from the reference sequences (RefSeq) available in NCBI as of July 2012.
[c]Tick, *Ixodes scapularis*, proteins were from the reference sequences (RefSeq) available in NCBI as of July 2012. *I. scapularis* is the closest relative to spiders with a sequenced genome.
[d]Only one tick protein with significant BLASTX alignment was retained per AT.
[e]Proportion of ATs that align to fly proteins, that aligned to more than one fly protein without overlap.
[f]CEGMA represents a database of Core Eukaryotic Genes that are conserved in all eukaryotes and should be expressed in all tissues [7].
[g]Black widow cDNAs and genes were compiled from GenBank and our personal databases. A non-redundant set of 999 sequences was constructed with CAP3.
