# Supplementary material for: Multi-tissue transcriptomics of the black widow spider reveals expansions, co-options, and functional processes of the silk gland gene toolkit
Source: BMC Genomics. 2014 Jun 11;15(1):365. doi: 10.1186/1471-2164-15-365 (PMC4200122; doi:10.1186/1471-2164-15-365)
Supplement: Supplementary file 2 — Additional file 2: Additional resources for the consequences of removing non-unique transcripts (Table S1), silk gland-specific gene family evolution (Table S2), and homologs of spider-specific genes (Tables S3-S5). (PDF 131 KB) [file 12864_2013_6107_MOESM2_ESM.pdf]

SUPPLEMENTARY TABLES

**Supplementary Table S1**. Proportion of raw sequencing reads that aligned to the assembled transcripts (ATs) in the Trinity derived transcriptome before and after removing redundant amino acid sequences (unique assembled transcripts, UATs) from each tissue type.		
Library	% Mapped to Original ATs	% Mapped to UATs
Cephalothorax	80.30%	73.85%
Silk glands	84.96%	86.24%
Venom glands	85.95%	81.32%

Supplementary Table S2. Observed versus expected evolutionary shifts for black widow assembled transcripts between generally expressed (non-Silk) and silk-specific expression (Silk).

Family ID[a]	SSTs[b]	nSSTs[c]	Observed non-Silk to Silk[d]	Expected non-Silk to Silk[e]	Observed Silk to non-Silk[d]	Expected Silk to non-Silk[e]
1	5	40	5	4.75+0.44	2	2.86+1.14
2	2	35	2	1.96+0.20	0	1.12+0.73
3	27	2	2	1.12+0.82	2	1.91+0.29
4	3	26	2	2.86+0.38	0	1.61+0.97
6	9	20	7	6.80+1.37	3	5.17+2.27
7	13	15	2	7.90+2.73	6	5.98+2.33
9	13	9	0	6.95+2.77	3	4.89+1.52
10	2	20	2	1.94+0.24	0	1.15+0.76
11	2	19	2	1.95+0.22	2	1.11+0.67
12	13	8	0	5.64+2.76	3	4.87+1.52
13	4	16	3	3.42+0.67	0	2.53+1.32
14	3	15	2	2.69+0.54	1	1.85+1.08
15	11	7	8	5.17+2.51	4	4.05+1.51
16	14	3	2	2.22+2.17	3	2.71+0.59
17	4	12	1	3.26+0.85	3	2.20+1.46
21	2	11	1	1.89+0.31	3	1.03+0.76
26	5	6	0	3.07+1.30	3	2.64+1.34
27	5	5	3	2.60+1.26	5	2.24+1.17
28	5	5	1	2.75+1.23	1	2.06+1.13
33	3	6	3	2.30+0.77	0	1.72+1.11
35	2	6	2	1.66+0.50	0	1.28+0.87
37	2	5	0	1.40+0.67	4	1.31+0.93

38	3	4	1	1.92+0.88	0	1.57+0.92
40	4	3	0	2.00+1.09	2	1.65+0.86
47	2	4	2	1.37+0.68	0	1.29+0.77
51	4	2	2	1.39+0.97	2	1.31+0.69
52	2	4	0	1.37+0.66	2	1.24+0.83
53	2	3	1	1.29+0.73	0	1.20+0.82
55	3	2	0	1.29+0.95	2	1.18+0.67
56	3	2	0	1.17+0.92	2	1.18+0.74
59	2	3	2	1.26+0.71	0	1.12+0.78
63	2	3	0	1.34+0.68	2	1.03+0.78
76	2	2	2	1.15+0.70	1	0.86+0.71
82	2	2	2	1.02+0.70	1	0.94+0.72
83	2	2	0	1.06+0.71	2	0.96+0.70
AVG	3.85	9.34	1.77	2.62+1.00	1.82	2.05+1.01

[a]Family IDs were assigned to clusters generated with BLASTCLUST (see main document).
[b]silk gland-specific transcripts using the 2.5% tail cutoff (see Additional File 1, Figure S1)
[c]non-silk gland-specific transcripts using the 2.5% tail cutoff
[d]Observed state switches within the PhyML gene tree, with the interior nodes annotated as either silk-specific or not silk-specific using discrete ML ancestral state reconstruction.
[e]Mean and standard deviation of the expected state switches within 100 random phylogenetic gene trees, with the interior nodes annotated as either silk-specific or not silk-specific using discrete ML ancestral state reconstruction.

Supplementary Table S3. Black widow unique assembled transcripts (UATs) with best BLASTX hit to *Nephila clavipes* Aggregate Spider Glue 1 (GenBank:ACI41238.1).

UAT	Transcript Length	Protein Length	Cephalothorax eCPM	Silk eCPM	Venom eCPM	E-Score
Contig26361	1666	377	27.63	0.00	0.08	8.00E-40
Contig21981	436	139	0.60	0.00	0.13	3.00E-22
silk_Contig684	469	126	5.28	2.99	0.00	1.00E-22
Contig26359	1777	413	41.59	7.79	0.16	3.00E-39
ceph_comp63067_c0_seq1	234	77	0.00	0.00	0.00	6.00E-11
silk_Contig682	1240	383	44.53	14.64	0.32	3.00E-45
Contig26360	1702	389	0.07	0.00	0.00	9.00E-40
Contig12095	1178	322	4.16	1.64	0.10	3.00E-18
Contig21628	512	157	0.26	1.56	0.00	1.00E-06
ceph_comp63067_c1_seq1	320	106	0.00	0.09	0.00	9.00E-17
ceph_comp92413_c0_seq6	1465	310	3.00	0.00	0.17	2.00E-34
Contig10398	1064	348	0.36	1.90	0.18	9.00E-17
ceph_comp92413_c0_seq5	1501	322	120.21	0.00	0.00	2.00E-34
Contig26362	1573	346	29.69	1.59	1.22	5.00E-40

Supplementary Table S4. Black widow unique assembled transcripts (UATs) with best BLASTX hit to *Latrodectus hesperus* Aggregate gland Silk Factor 1 (GenBank:AFP57565.1)

UAT	Transcript Length	Protein Length	Ceph eCPM	Silk eCPM	Venom eCPM	E-Score
silk_Contig525	1102	367	5.0	100412.9	2.0	5E-74
silk_comp17078_c1_seq1	436	98	0	19859.7	0	1E-22
Contig17258	478	159	3.0	358949.9	2.0	5E-19
silk_Contig646	375	124	0	30987.6	0	1E-07
Contig13471	2670	763	510.0	15.0	84.0	5E-07
silk_Contig644	955	135	0	74294.7	0	3E-04
silk_Contig643	733	85	0	48653.4	0	4E-04

Supplementary Table S5. Black widow unique assembled transcripts (UATs) with best BLASTX hits to published spidroin N or C-terminal regions.

UAT	Spidroin[a]	Terminal Region	Repeat Size[b]	Nucleotide Identity[c]
Lhsilk_Contig1000	Flag	C	279	NA
silk_comp16631_c0_seq2	MaSp1	C	45	99%
C_Lh_Contig384	MaSp1	C	45	96%
Lhsilk_comp17907_c0_seq1	MaSp2	C	80	99%
Lhsilk_comp15685_c0_seq1	MaSp1	C	226	77%
Lhsilk_comp17612_c0_seq2	MiSp	C	136	100%
Lhsilk_comp17612_c0_seq1	MiSp	C	179	85%
LhContig1319	TuSp1	C	251	98%
C_LhContig301	AcSp1	C	674	99%
C_LhContig48	PySp	C	286	97%
C_Lhsilk_comp12682_c1_seq1	MaSp1	N	39	74%
C_Lhsilk_Contig691	MaSp1	N	87	99%
C_Lhsilk_comp10331_c0_seq1	MaSp1	N	16	99%
Lhsilk_comp8200_c0_seq1	AcSp1	N	27	100%
Lhsilk_comp18043_c0_seq1	TuSp1	N	136	99%
Lhsilk_comp15188_c0_seq1	MaSp2	N	0	100%
Lhsilk_Contig933	MiSp	N	140	97%
Lhsilk_comp15188_c1_seq1	MaSp2	N	78	100%
LhContig499	Flag	N	479[d]	NA
Lhsilk_comp10420_c1_seq1	Flag	N	19	NA

[a]Spidroin determined by lowest BLASTX E-score to all published spidroin sequences.
[b]Number of amino acids outside the published terminal regions in [1, 2].
[c]to most similar published *L. hesperus* spidroin encoding gene.
[d]Sequence terminated in stop codon.

References

1. Garb JE, Ayoub NA, Hayashi CY: **Untangling spider silk evolution with spidroin terminal domains.** *BMC evolutionary biology* 2010, **10**(1)**:**243-243.
2. Ayoub NA, Garb JE, Kuelbs A, Hayashi CY: **Ancient properties of spider silks revealed by the complete gene sequence of the prey-wrapping silk protein (AcSp1).** *Mol Biol Evol* 2013, **30**(3)**:**589-601.
