# Supplementary material for: Multi-tissue transcriptomics of the black widow spider reveals expansions, co-options, and functional processes of the silk gland gene toolkit
Source: BMC Genomics. 2014 Jun 11;15(1):365. doi: 10.1186/1471-2164-15-365 (PMC4200122; doi:10.1186/1471-2164-15-365)
Supplement: Supplementary file 3 — Additional file 3: Additional resources for identifying possible bacterial contamination (Figure S2-4) and silk gland-specific transcript identification (Figure S1,S5). (PDF 214 KB) [file 12864_2013_6107_MOESM3_ESM.pdf]

Supplementary Figures

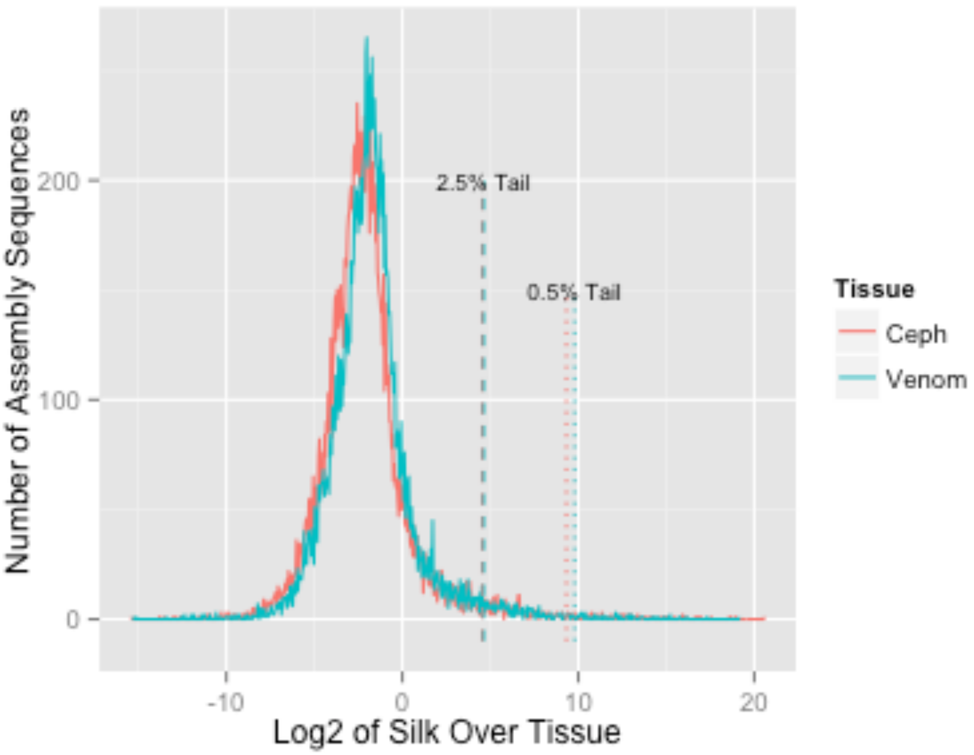

Figure S1. Distribution of log2 of silk:cephalothorax (blue) and silk:venom (orange) eCPM ratios for black widow unique assembled transcripts (UATs). The cutoffs for both the 2.5% tail (dashed line) and the 0.5% tail (dotted line) are shown for both the silk:cephalothorax (blue) and the silk:venom (orange). UATs with eCPM < 1 in all tissues were excluded from analysis. UATs with eCPM = 0 in venom glands or cephalothorax were excluded from calculation of ratios.

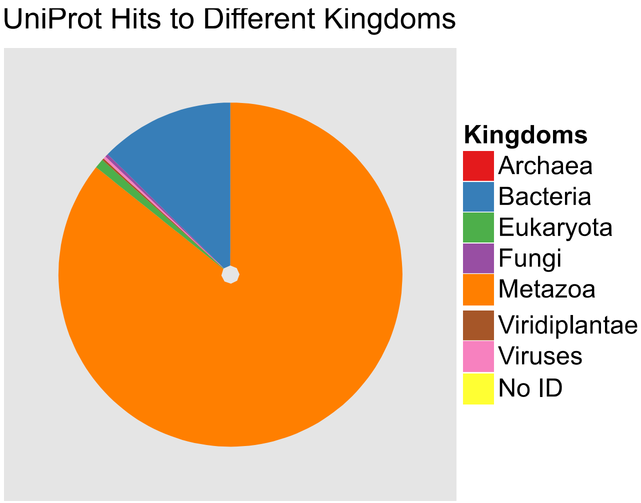

Figure S2. Kingdom of most similar UniProtKB protein of annotated black widow assembled transcripts. For all the full assembled transcripts, the most similar UniProt sequence was determined using lowest E-scores, with a maximum allowed E-score of 1e-5. The kingdom was assigned using the UniProt taxonomic ID.

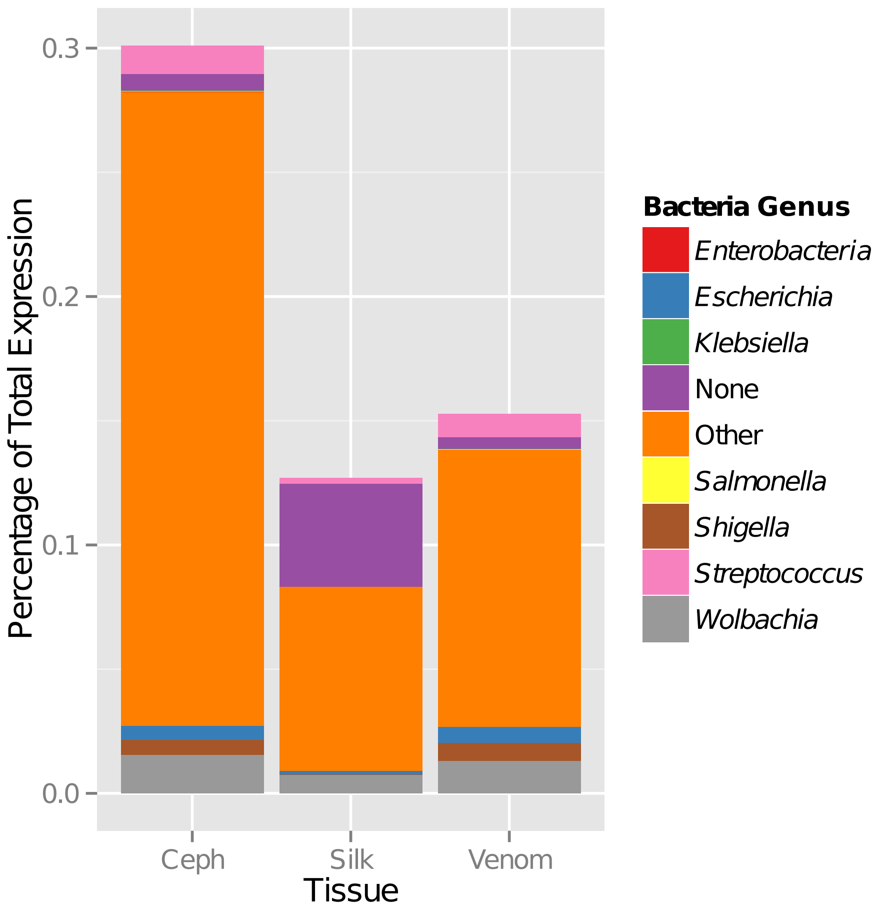

Figure S3. Percent expression contributed by suspected bacterial-derived unique assembled transcripts (UATs). Expression is broken down by the genus of bacteria. The genera were assigned using the UniProt taxonomic ID of the best BLASTX match to assembled transcripts.

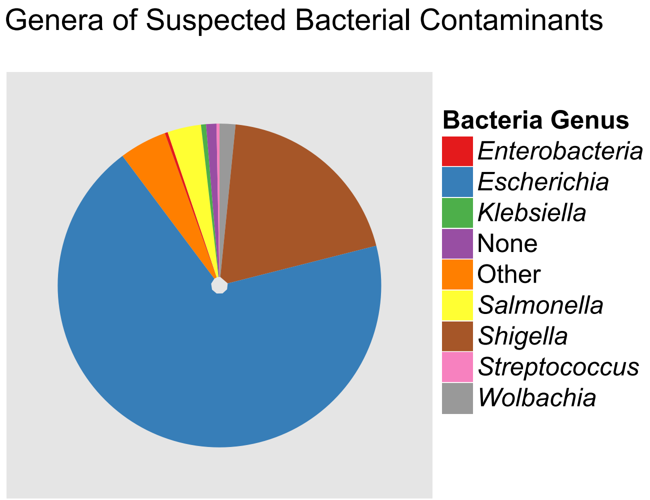

Figure S4. Proportion of bacterial genera for suspected bacterial-derived unique assembled transcripts (UATs). The most similar UniProtKB sequence was determined using lowest E-scores, with a maximum allowed E-score of 1e-5. The genus for each transcript was assigned using the UniProt taxonomic ID. Only UATs with a bacterial sequence as the most similar annotated UniProt hit were included.

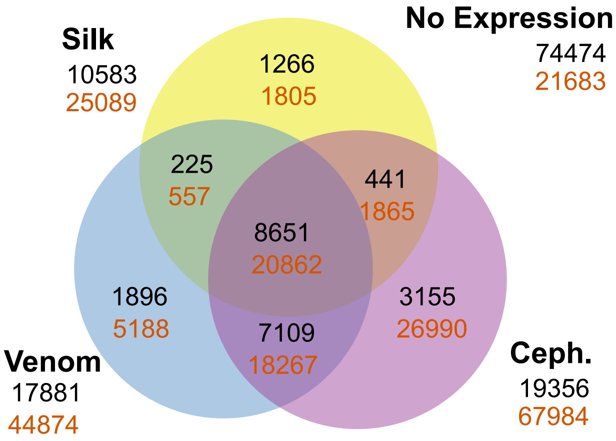

Figure S5. Number of unique assembled transcripts (UATs) expressed in each black widow tissue. The number of UATs that are expressed in each tissues either above 1 eCPM (shown in black) or above 0 eCPM (shown in orange) in the silk glands (yellow circle), the cephalothorax (pink circle), and the venom gland (blue circle).
